# Bidirectional effects of speech and gesture: evidence from a novel levels-of-congruence paradigm

**DOI:** 10.3389/fpsyg.2026.1851588

**Published:** 2026-08-19

**Authors:** Laura Pissani, Vera Demberg

**Affiliations:** Department of Language Science and Technology, Saarland University, Saarbrücken, Germany

**Keywords:** co-speech gestures, gesture comprehension, integrated-systems hypothesis, metaphor comprehension, speech-gesture integration

## Abstract

**Introduction:**

Previous research has shown that speech and gesture are integrated during comprehension, with differences in response times and electrophysiological activity observed between incongruent and congruent gesture—speech pairings. Beyond these incongruency effects, it remains unclear whether comprehenders use gestures to adapt or extend conventional interpretations for novel communicative purposes.

**Method:**

The current study examined the bidirectional influence of speech and gesture by employing a novel levels-of-congruence paradigm, in which the same metaphor (e.g., *Men are oaks*) was paired with a gesture depicting either its preferred interpretation (e.g., men are robust) or a less preferred but possible interpretation (e.g., men are tall). In Experiment 1, participants rated the meaningfulness of preferred and possible interpretations across three gesture conditions (gesture-of-preferred, gesture-of-possible, non-gestured). In Experiment 2, participants selected a single interpretation under the same conditions. In Experiment 3, participants viewed clips of an actor producing the same gesture and utterance combination (e.g., flexing both arms while saying *Men are oaks*) in one of three multimodal conditions: no additional cues (muted, blurred lips), only lip movements (muted, visible lips), or audible speech. Then they rated how well the gesture matched the gestured interpretation (e.g., *robust*), an alternative interpretation consistent with the speech but not the gesture (e.g., *tall*), and an unrelated interpretation (e.g., *hikers*).

**Results:**

In Experiments 1 and 2, participants consistently rated the preferred interpretation as the most meaningful and selected it most often across all gesture conditions. However, gestures depicting the possible interpretation significantly increased both the meaningfulness ratings and the likelihood of being selected of the possible interpretation. In Experiment 3, the presence of speech cues significantly increased ratings for the alternative interpretation compared with the condition without additional cues.

**Discussion:**

These findings demonstrate bidirectional interactions between speech and gesture during metaphor comprehension. Gestures constrained metaphor interpretation, whereas speech broadened the range of interpretations attributed to gestures. Overall, the results support the integrated-systems hypothesis by showing that speech and gesture jointly contribute to the construction of meaning.

## 1 Introduction

Language and gesture are argued to form an integrated system, in which each modality contributes to the underlying meaning of utterances (McNeill, 1992). As McNeill (2005) puts it, gestures “are integrated with linguistic content into growth points and appear, to the speaker, as an unbroken package of semiosis with it.” The notion of an *unbroken package* implies a bidirectional relationship between speech and gesture. Not only do gestures influence how language is comprehended, but language also affects how gestures are processed.

The relationship between speech and gesture is particularly evident in how gesture production is shaped by the linguistic structures of a given language. Kita and Özyürek (2003) observed that English speakers, who can encode arc motion in a verb (e.g., *to swing*), tend to produce arc-shaped gestures when retelling events involving this movement. In contrast, Turkish and Japanese speakers, whose languages lack a verb that encodes arc motion, rely on more general verbs (e.g., *to go, to jump, to fly*) and produce predominantly straight gestures in their retellings. Similarly, English speakers who encode manner and trajectory in a single construction (e.g., *to roll down*) tend to produce a single gesture to describe the movement, whereas Turkish and Japanese speakers, who require two clauses to express the same concept (e.g., *to descend by rolling*), tend to represent these aspects in separate gestures.

Beyond cross-linguistic variation, experimental work has also demonstrated the close relationship between speech and gesture. Kelly et al. (1999, 2010b) proposed the integrated-systems hypothesis, according to which speech and gesture are *mutually* and *obligatorily* integrated during comprehension. To test this hypothesis, Kelly et al. (2010b) used a priming task in which primes were videos of actions (e.g., a person chopping vegetables) and targets were videos of an actor gesturing while speaking. The relationship between prime and target was manipulated separately for speech and gesture using a levels-of-incongruence paradigm. In this paradigm, either speech or gesture was weakly incongruent (e.g., a “cut” gesture or the actor saying *cut*) or strongly incongruent (e.g., a “twist” gesture or the actor saying *twist*) relative to the prime. Participants were asked to respond “yes” or “no” to whether the target was related to the prime, and responses in the incongruent conditions were compared to a congruent baseline (e.g., a “chop” gesture and the actor saying *chop*). Results indicated that participants made more errors when either speech or gesture was strongly incongruent compared to weakly incongruent, and this effect was similar across modalities. These findings support the integrated-systems hypothesis by showing that speech and gesture influence one another. This influence appears to be obligatory, as participants were unable to ignore gestures even when instructed to attend only to the verbal content.

Several other studies have further examined the influence of gestures on language comprehension by employing an incongruency paradigm (Cornejo et al., 2009; Beattie and Sale, 2012; Kelly et al., 2010a, 2015; Wu and Coulson, 2005; Özer et al., 2026, see for a review Kananda Arachchige et al., 2021). Similar to Kelly et al.'s study, utterances are paired with gestures that are either congruent or incongruent with their content. Differences are measured behaviorally–using response times and error rates (e.g., Beattie and Sale, 2012)–or via electrophysiological brain activity (e.g., Cornejo et al., 2009), and indicate the extent to which speech and gesture are integrated during comprehension.

At the behavioral level, Beattie and Sale (2012) examined how people integrate speech and gesture information in semantic and social judgments. Participants watched videos of a person uttering a message (e.g., …*we are very close*) while producing either a matching gesture (hands moving rapidly toward each other until an inch apart) or a mismatching gesture (hands stopping six inches apart), and subsequently rated their interpretation of the message (e.g., Did they have a good relationship?) and their impressions of the person (e.g., How much do you instinctively like this person?) on a five-point scale. Participants who observed mismatching gestures tended to adjust their responses according to the gesture and rated the speaker as less likable and trustworthy, suggesting that gesture mismatches affect how messages are perceived.

At the neural level, Cornejo et al. (2009) investigated whether gestures influence the comprehension of metaphors. They compared the electrophysiological brain activity of participants watching videos in which gestures were either congruent or incongruent with the figurative content of the speech (e.g., *Those men are oaks*). Greater negativity for incongruent gestures emerged at the onset of the gesture stroke (approximately 400 ms after the onset of the critical word *oaks*), suggesting that information conveyed by gestures is integrated early during metaphor comprehension.

This incongruence paradigm allows researchers to investigate the nature and time course of gesture-speech integration. Specifically, it can reveal whether mismatches between speech and gestures are detected during comprehension, reflected in longer reaction times or more pronounced electrophysiological patterns. For instance, Kelly et al. (2010a) found that participants took longer and produced larger N400 amplitudes when gestures were incongruent with speech, even in a task that did not require attending to the gestures (i.e., identifying the speaker's gender), consistent with the automatic integration of speech and gesture. However, this paradigm may be less suited for examining how gestures could modulate or even shift the interpretation of an utterance in a novel way. In the present study, we used a novel levels-of-*congruence* paradigm, in which the verbal content consists of metaphors that afford more than one interpretation, and gestures match either the preferred interpretation or a less preferred but possible interpretation. Since both gestures are congruent with viable interpretations of the utterance, this design allowed us to examine whether comprehenders use gestures to adapt or extend conventional interpretations for novel communicative purposes.

A further aim of this study was to investigate the influence of verbal content on *gesture* comprehension. Most research has focused on the role of gestures in language comprehension, reporting their benefits for disambiguating meaning (Holle and Gunter, 2007; Debreslioska and Gullberg, 2020), conveying additional semantic information (Holler et al., 2009), deepening understanding (Kang and Tversky, 2016; Kang et al., 2013), improving memory performance (Iani and Bucciarelli, 2017; Straube et al., 2009; Guo et al., 2024), and predicting upcoming words (Reimer et al., 2024; Ter Bekke et al., 2020). However, relatively few studies have examined how gesture comprehension is influenced by linguistic and contextual factors (e.g., Huang et al., 2025; Kelly et al., 1999).

### 1.1 The current study

In the present study, we used metaphorical expressions and co-speech gestures to investigate whether the interpretation of a given utterance is influenced by the gestures paired with it, and conversely, whether the interpretation of gestures is influenced by the accompanying speech.

The speech portion consisted of metaphorical expressions of the form *X is Y*, in which the vehicle *Y* predicates properties of the topic *X*. For example, in the expression *Pets are kids*, several properties of kids can be attributed to pets, such as being loving, playful, dependent, and innocent. The gesture accompanying each expression visually depicted one of these properties, for example, both hands held together at chest level to convey love. Because these gestures represent the figurative content of the metaphor, namely that pets are loving, rather than depicting the concrete referent *kids*, they are metaphoric gestures, as they convey an abstract concept (McNeill, 1992). It is important to note that some degree of iconic resemblance may nevertheless arise (see Cornejo et al., 2009). For example, in *Roads are snakes*, where the property predicated is winding, a hand moving in a curving motion may resemble the shape of a snake. However, because the gesture represents the metaphorical construal of the road rather than a concrete snake, it can still be considered metaphoric rather than iconic (see Khatin-Zadeh et al., 2022).

Given that most metaphors allow for multiple interpretations, we used metaphorical expressions to develop a levels-of-*congruence* paradigm. In this paradigm, the same utterance was paired with gestures corresponding either to its preferred interpretation or to a less preferred but still possible one. For instance, the same metaphorical expression (e.g., *Men are oaks*) was paired with a gesture matching its preferred interpretation (men are robust), a gesture matching a less preferred but possible interpretation (men are tall), or no gesture.

We combined the levels-of-congruence paradigm with a meaningfulness rating task (Experiment 1), in which participants evaluated preferred, possible, and impossible interpretations (*robust, tall*, and *hikers*) of the same utterance across three gesture conditions (gesture-of-preferred, gesture-of-possible, non-gestured), and a forced-choice task (Experiment 2), in which participants selected a single interpretation under the same gesture conditions. We further employed this approach to investigate how speech cues influence gesture comprehension (Experiment 3) by presenting the same ‘robust' gesture in three multimodal conditions (no additional cues, lip movements only, audible speech) and asking participants to rate how well it matched the gestured interpretation (*robust*), an alternative interpretation related to the speech but not the gesture (*tall*), and an unrelated interpretation (*hikers*).

## 2 Experiment 1: meaningfulness rating task

Experiment 1 had two main goals. The primary goal was to investigate whether participants incorporated information from gestures in their judgments by examining whether meaningfulness ratings for two plausible interpretations of a metaphor were influenced by the accompanying gesture.

The secondary goal was to obtain meaningfulness ratings for each interpretation of the metaphor from the non-gestured videos, in order to determine which interpretation was preferred and which was less preferred but still possible when no gesture was present.

### 2.1 Method

#### 2.1.1 Participants

A total of 60 native English speakers (age range: 21–70 years; M = 44.78, SD = 12.71; 25 female) participated in the study via Prolific.^1^ The study lasted approximately 15 minutes, and participants were compensated with £2.50. All participants were born in the United States and were residing there at the time of the study.^2^

#### 2.1.2 Materials

We selected a set of 28 metaphors of the form *X is Y* (e.g., *Men are oaks*) from Roncero and de Almeida (2015) and Cornejo et al. (2009). The metaphors were chosen to vary in familiarity and to allow at least two plausible interpretations that could be easily represented through gesture. Specifically, we chose 22 expressions from the set of 84 expressions normed by Roncero and de Almeida (2015) and 6 expressions from the set of 24 used in Cornejo et al. (2009)'s study.

Then, we identified two plausible interpretations for each metaphor, along with an impossible interpretation to serve as a distractor. For items obtained from Roncero and de Almeida (2015), we determined two interpretations based on the properties of the topic-vehicle pairing provided in their norms. For instance, for *Anger is fire*, we chose *hot* and *destructible* from the set of *dangerous, hot, red, scary, burning, hurtful, destructive*, and *deadly*, as these captured different aspects of the metaphor and were easy to gesture. For items obtained from Cornejo et al. (2009), we determined two interpretations based on properties commonly associated with the vehicle term. For example, oaks are commonly associated with strength, sturdiness, or large size, so we chose *robust* and *tall*, as these also represented different aspects of the metaphor and were easy to gesture. Afterwards, we conducted a pilot study (*N* = 15) with open-ended questions (see Supplementary Materials), which we used to refine some interpretations before the first recording session.

**Figure 1:**
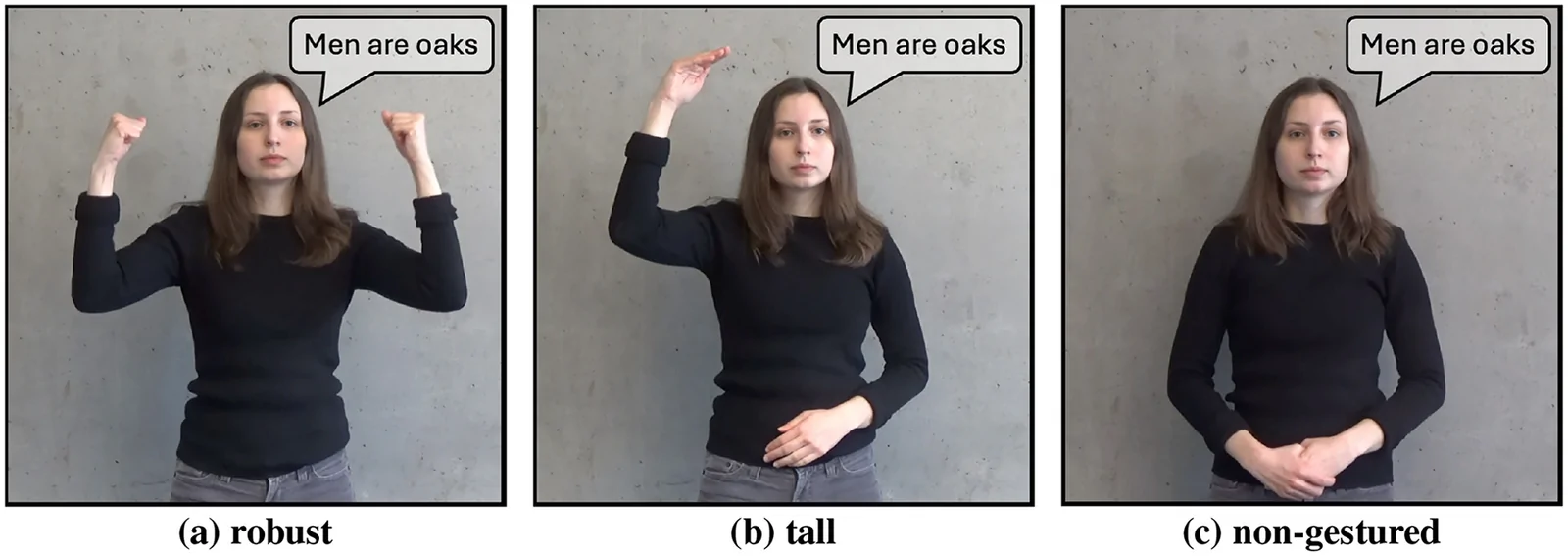
Video frames showing gestures for the metaphor *Men are oaks*, corresponding to the two plausible interpretations of the expression and a non-gestured condition. **(a)** Robust. **(b)** Tall. **(c)** Non-gestured.

Each metaphor was recorded in three versions: with a gesture related to one interpretation (Figure 1a), with a gesture related to the other interpretation (Figure 1b), and without any accompanying gesture (Figure 1c). During the recording process, four metaphors were excluded due to the difficulty of gesturing their figurative interpretations (i.e., *Christians are salt, Debt is disease, Science is politics*, and *Winter is death*), which resulted in a final set of 24 metaphors, each associated with two interpretations.^3^

The actor was a native English speaker and was the same for all recordings. All gestures were agreed upon by the first author and the actor and were practiced before recording. Gestures involved only the hands, avoiding any meaningful facial movements. Recording was done with audio using a SONY HDR-CX405 camera. Post-recording, the videos were edited to remove background noise and exported in 4K resolution with HD image enhancement.

A total of 72 videos were recorded, including 48 with gestures (M = 5.24 s, SD = 0.56) and 24 without gestures (M = 5.37 s, SD = 0.51). These videos were divided into three lists, with gesture condition treated as a within-subjects factor, each containing 16 videos with gestures and 8 without gestures. To balance the proportion of gestured and non-gestured items, 24 filler videos were added (8 with gestures and 16 without gestures), resulting in a total of 48 trials per list.

##### 2.1.2.1 Gesture norms

We conducted a first norming study to assess whether the gestures conveyed their intended interpretation and were not associated with alternative or unrelated interpretations. To ensure that participants evaluated only the gestures and not the verbal content, the audio was muted and the speaker's lips were blurred.

For example, for the metaphor *Men are oaks*, the actor performed two gestures, one with both arms flexed to indicate robustness (Figure 1a) and one with a hand raised above the head to indicate tall stature (Figure 1b). Each video was presented with three written interpretations (gestured, alternative, and unrelated) displayed below it. For the video of the “robust” gesture, *robust* was labeled as the gestured interpretation (*robust*), *tall* was the alternative interpretation, and *hiker* was the unrelated interpretation. For the video of the ‘tall' gesture, the same three interpretations were presented, but the internal labeling of *gestured* and *alternative* was reversed (see Supplementary Table 1).

Participants (*N* = 50) received the following instructions: *In this task, you will watch a series of very short video clips. In each clip, a woman will be gesturing. Your task is to rate how well the gesture represents three given interpretations on a scale from 1 (not at all) to 5 (very much)*. Each trial also included a reminder to rate how well the gesture corresponded to each interpretation, ensuring that attention remained focused on the gestures. Each participant completed 24 critical trials, seeing only one gesture per metaphor. Four additional attention trials featuring universally recognizable gestures were included to ensure participants were attending to the task (e.g., if the gesture depicted a heart, the interpretation *heart* was expected to receive the highest rating). A SKIP option was provided for videos in which participants were unable to view the gesture clearly.

Supplementary Table 2 presents the mean ratings for the gestured, alternative, and unrelated interpretations for each gesture associated with the metaphor. Overall, the gestured interpretation was rated significantly higher than both the alternative interpretation (t_(23)_ = 11.48, *p* < 0.001, 95% CI [1.56, 2.25]) and the unrelated interpretation (t_(23)_ = 13.48, *p* < 0.001, 95% CI [1.88, 2.56]).

##### 2.1.2.2 Familiarity norms

We conducted a second norming study to obtain familiarity ratings for each expression in all gesture conditions. This served two purposes. First, since unfamiliar metaphors are processed differently than familiar metaphors (e.g., Blasko and Briihl, 1997; Blasko and Connine, 1991, 1993; Damerall and Kellogg, 2016; Stamenković et al., 2019), familiarity ratings of non-gestured videos were included as a covariate in all analyses to account for this variation. Second, familiarity ratings were compared when the expression was presented in the gesture-of-preferred vs. gesture-of-possible conditions to examine whether the perceived familiarity of an expression was influenced by the gesture paired with it.

Participants (*N* = 60) watched the videos and were asked to report the extent to which they had heard or read that expression in the past using a scale from 1 (not familiar at all) to 7 (very familiar). In the videos, the actor either produced a gesture conveying one of the two interpretations of the metaphor or did not gesture. Each participant completed 24 critical trials, seeing only one condition per metaphor, and 24 fillers to balance the number of gestured and non-gestured videos.

Supplementary Table 3 presents the mean familiarity ratings for each metaphor in the non-gestured condition. Overall, familiarity ratings did not differ significantly between utterances paired with gestured versus non-gestured presentations, (t_(23)_ = −2.02, *p* = 0.06, 95% CI [−0.28, 0.00], or between utterances paired with gestures conveying the preferred versus possible interpretations, (t_(23)_ = 0.43, *p* = 0.67, 95% CI [−0.21, 0.33].

#### 2.1.3 Procedure

The meaningfulness rating task was implemented in LingoTurk (Pusse et al., 2016). Immediately after watching each video, participants were presented with the three interpretations in random order (two plausible and one implausible). Their task was to rate how well each interpretation conveyed the meaning of the expression on a five-point scale. Participants were not informed that some videos included gestures while others did not, allowing us to examine whether gestures naturally influenced their judgments.

#### 2.1.4 Statistical analysis

We fitted Bayesian ordinal regression models with a cumulative family and a logit link using the brms package (Bürkner, 2017, 2018, 2021) in R version 2026.01.0 (R Core Team, 2024). Model 1 was specified to predict changes in meaningfulness ratings as a function of (gesture-of-preferred, gesture-of-possible, non-gestured), interpretation (preferred, possible, impossible), and familiarity, with varying intercepts for participants and items and varying item-specific slopes for interpretation × gesture combinations:


$$\begin{array}{lll} \text{rating} & \sim & \text{interpretation}*\text{gesture}+\text{familiarity}+\left(1|\text{participant}\right)\\ +\left(1+\text{interpretation}:\text{gesture}|\text{item}\right) \end{array}$$

Model 1a additionally examined whether the effects of gesture and interpretation varied as a function of familiarity by including all two-way and three-way interactions among these predictors:


$$\begin{array}{lll} \text{rating} & \sim & \text{interpretation}*\text{gesture}*\text{familiarity}+\left(1|\text{participant}\right)\\ +\left(1+\text{interpretation}:\text{gesture}|\text{item}\right) \end{array}$$

Models 1 and 1a used weakly informative priors, *N*(0, 1), for all regression coefficients. These included the main effects of gesture, interpretation, and familiarity, as well as all interaction terms specified in each model. All models converged well ($\hat{R}=1.0$), and posterior predictive checks indicated that the models accurately captured the distribution of the observed data.

### 2.2 Results

This section first presents descriptive statistics for the meaningfulness rating task, followed by the regression analysis examining whether gestures influenced metaphor comprehension. The change in meaningfulness ratings was modeled as a function of gesture (gesture-of-preferred, gesture-of-possible, non-gestured) and interpretation (preferred, possible, impossible).

We hypothesized an interaction between gesture and interpretation. Specifically, when the gesture matched the preferred interpretation of the metaphor, meaningfulness ratings were expected to be higher for the preferred interpretation than for the possible interpretation. Conversely, when the gesture matched the possible interpretation, meaningfulness ratings were expected to increase for the possible interpretation, and be higher than or equal to ratings for the preferred interpretation.

#### 2.2.1 Descriptive statistics

##### 2.2.1.1 Interpretation

We first identified the preferred and possible interpretations for each metaphor, independent of gesture. For each metaphor, we calculated the mean rating for each interpretation and labeled the one with the higher meaningfulness rating as *preferred* and the other one as *possible*.

To confirm that the resulting preferred, possible, and impossible interpretations were statistically differentiated in terms of perceived meaningfulness, we conducted paired-samples t-tests. As expected, ratings of preferred interpretations (M = 4.10, SD = 0.43) were significantly higher than those of possible interpretations (M = 3.22, SD = 0.57), *t*(23) = 7.83, *p* < 0.001, 95% CI [0.65, 1.12]. Similarly, ratings of possible interpretations were significantly higher than those of impossible interpretations (M = 2.21, SD = 0.99), *t*(23) = 4.01, *p* < 0.001, 95% CI [0.49, 1.52].

Supplementary Table 3 presents the meaningfulness ratings for each interpretation. For example, for *Women are roses*, the preferred interpretation was beautiful (M = 4.6, SD = 0.8), the possible interpretation was fragrant (M = 3.0, SD = 0.9), and the impossible interpretation was red (M = 1.6, SD = 1.0).^4^

##### 2.2.1.2 Gesture

We then recoded the gestures for each metaphor based on whether they matched the preferred interpretation (gesture-of-preferred), the possible interpretation (gesture-of-possible), or if no gesture was produced (non-gestured). Table 1 presents the average meaningfulness ratings for each interpretation (preferred, possible, impossible) as a function of gesture (gesture of preferred, gesture of possible, non-gestured).

**Table 1:** Meaningfulness ratings for preferred, possible, and impossible interpretations as a function of gesture.

Gesture	Preferred	Possible	Impossible
Gesture-of-preferred	4.28 (0.97)	3.06 (1.41)	2.20 (1.42)
Gesture-of-possible	3.89 (1.29)	3.68 (1.31)	2.09 (1.40)
Non-gestured	4.12 (1.12)	3.22 (1.35)	2.22 (1.45)

#### 2.2.2 Regression model

The regression coefficients from Model 1 are summarized in Table 2.^5^ Across all gesture conditions, the preferred interpretation received higher meaningfulness ratings than both the possible and impossible interpretations. However, meaningfulness ratings for the possible interpretation were higher in the gesture-of-possible condition than in both the gesture-of-preferred condition ($\hat{\beta }=1.37$, 95% CrI[0.73, 1.96]) and the non-gestured condition ($\hat{\beta }=1.08$, 95% CrI[0.55, 1.61]). Moreover, this pattern was reversed for the preferred interpretation, as meaningfulness ratings were lower in the gesture-of-possible condition than in the gesture-of-preferred condition ($\hat{\beta }=-0.43$, 95% CrI[-0.81, -0.02]) and in the non-gestured condition ($\hat{\beta }=-0.36$, 95% CrI[-0.69, -0.02]).

**Table 2:** Model 1 regression coefficients predicting the log-odds of higher meaningfulness ratings by gesture and interpretation.

Parameter	$\hat{\beta }$	95% CrI
Fixed effects		
Interpretation (possible)	-1.83	[-2.28, -1.34]
Interpretation (impossible)	-3.17	[-3.84, -2.43]
Gesture (non-gestured)	-0.08	[-0.41, 0.28]
Gesture (gesture-of-possible)	-0.43	[-0.81, -0.01]
Familiarity	0.11	[-0.08, 0.30]
Possible × non-gestured	0.26	[-0.25, 0.74]
Impossible × non-gestured	0.01	[-0.58, 0.57]
Possible × gesture-of-possible	1.37	[0.73, 1.96]
Impossible × gesture-of-possible	0.10	[-0.54, 0.69]
Random effects		
*Item*		
Intercept	0.67	[0.39, 0.99]
Preferred × preferred gesture slope	0.51	[0.04, 1.07]
Possible × preferred gesture slope	0.71	[0.25, 1.18]
Impossible × preferred gesture slope	1.51	[1.10, 2.02]
Preferred × gesture-of-possible slope	0.70	[0.27, 1.16]
Possible × gesture-of-possible slope	0.91	[0.50, 1.39]
Impossible × gesture-of-possible slope	1.57	[1.15, 2.09]
Preferred × non-gestured slope	0.52	[0.06, 0.98]
Possible × non-gestured slope	0.49	[0.05, 0.91]
Impossible × non-gestured slope	1.70	[1.25, 2.26]
*Participant*		
Intercept	0.85	[0.70, 1.04]

**Figure 2:**
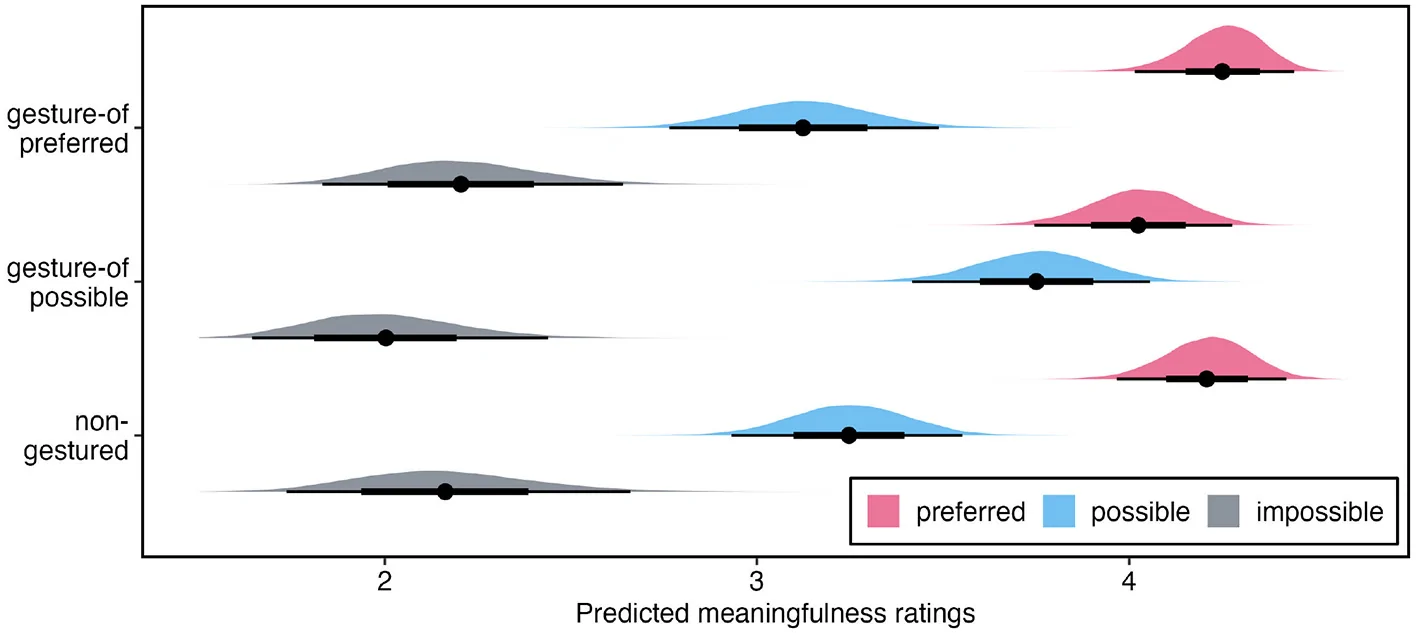
Predicted meaningfulness ratings for preferred, possible, and impossible interpretations across levels of gesture from Model 1.

Consistent with our hypotheses, when the gesture matched the preferred interpretation of the metaphor (e.g., robust for *Men are oaks*), meaningfulness ratings were higher for the preferred interpretation (*robust*) than for the possible interpretation (*tall*). In contrast, when the gesture matched the possible interpretation (e.g., tall for *Men are oaks*), meaningfulness ratings for the preferred and possible interpretations were more similar (see Figure 2).

#### 2.2.3 Follow-up analyses

The following analyses examined whether the interaction between gesture and interpretation varied as a function of familiarity. Highly familiar expressions may be more strongly associated with their default interpretations, which may reduce the influence of gesture. In contrast, less familiar metaphors may be more flexible and easier to shift depending on gesture.

##### 2.2.3.1 Familiarity effects

Model 1a included familiarity as an interaction term rather than as a covariate, resulting in a three-way interaction among interpretation (preferred, possible), gesture (gesture-of-preferred, gesture-of-possible, non-gesture), and familiarity (ranging from 1.7 to 5.9). To simplify interpretation, the impossible interpretation was excluded, as it was not relevant to the familiarity effect.

The two-way and three-way interactions were small, and the 95% credible intervals included zero (see Table 3), providing no clear evidence that the effect of gesture on interpretation varied reliably across levels of familiarity. This suggests that, within the observed range of familiarity, gesture influenced interpretation in a relatively stable manner rather than being modulated by how familiar an expression was.

**Table 3:** Model 1a regression coefficients predicting the log-odds of higher meaningfulness ratings by gesture, interpretation, and familiarity.

Parameter	$\hat{\beta }$	95% CrI
Fixed effects		
Interpretation (possible)	−1.28	[−2.34, −0.14]
Gesture (non-gestured)	0.25	[−0.53, 1.05]
Gesture (gesture-of-possible)	−0.08	[−0.92, 0.80]
Familiarity	0.17	[−0.13, 0.49]
Possible × non-gestured	0.17	[−0.87, 1.19]
Possible × gesture-of-possible	0.76	[−0.53, 2.02]
Possible × familiarity	−0.23	[−0.54, 0.07]
Gesture-of-possible × familiarity	−0.14	[−0.38, 0.10]
Non-gestured × familiarity	−0.13	[−0.35, 0.09]
Possible × non-gestured × familiarity	0.08	[−0.21, 0.37]
Possible × gesture-of-possible × familiarity	0.24	[−0.12, 0.60]
Random effects		
*Item*		
Intercept	0.83	[0.52, 1.21]
Preferred × preferred gesture slope	0.47	[0.03, 1.05]
Possible × preferred gesture slope	0.82	[0.23, 1.35]
Preferred × gesture-of-possible slope	0.58	[0.08, 1.14]
Possible × gesture-of-possible slope	1.02	[0.52, 1.54]
Preferred × non-gestured slope	0.42	[0.02, 0.97]
Possible × non-gestured slope	0.56	[0.04, 1.02]
*Participant*		
Intercept	0.95	[0.78, 1.17]

### 2.3 Discussion

Experiment 1 examined whether participants integrated gestural information into their judgments. Meaningfulness ratings for each interpretation of a metaphor were modeled as a function of whether the accompanying gesture matched the preferred interpretation, the possible interpretation, or whether no gesture was present. Consistent with our predictions, when the gesture matched the preferred interpretation, ratings were higher for the preferred interpretation than for the possible one. However, when the gesture matched the possible interpretation, ratings for the preferred and possible interpretations were more similar—independent of participants' familiarity with the metaphor. These findings indicate that participants incorporated gestural information into their judgments, even though gestures were not mentioned in the task instructions and were not present in all trials. Nevertheless, gestural information was not sufficient to override the preferred interpretation of the metaphor, as the preferred interpretation received higher ratings across all gesture conditions.

Contrary to our expectations, we did not observe evidence that the effect of gesture on interpretation varied across different levels of familiarity. This finding is surprising, as one might assume that gestures would exert a stronger influence on the interpretation of less familiar expressions than on more familiar ones. One possible explanation is that familiarity primarily affects the strength of the perceived meaningfulness of an interpretation, whereas gesture influences which interpretation is favored. Under this account, familiarity and gesture contribute in an additive rather than interactive manner, resulting in independent effects.

Since participants in Experiment 1 evaluated each interpretation individually, they may have been able to assign similar ratings to both the preferred and possible interpretations. In Experiment 2, we examined whether this pattern would change when participants were required to select a single interpretation.

## 3 Experiment 2: forced-choice task

The primary goal of Experiment 2 was to examine whether gestural information is sufficient to shift the default (preferred) interpretation when participants are required to choose a single interpretation.

### 3.1 Method

#### 3.1.1 Participants

A total of 60 native English speakers (age range: 25–65 years; M = 46.00, SD = 9.46; 33 female), different from those in Experiment 1, participated in the study via Prolific. The study lasted approximately 10 minutes, and participants were compensated with £2.5. All participants were born in the United States and were residing there at the time of the study.

#### 3.1.2 Materials

The materials were identical to those used in Experiment 1.

#### 3.1.3 Procedure

The forced-choice task was also implemented using LingoTurk (Pusse et al., 2016). Similar to the rating task, participants watched videos of the same speaker, which were immediately followed by three interpretations. For this task, however, participants were prompted with the question *What did the speaker mean?* and the three interpretations were presented side by side; their task was to select the one they believed the speaker intended. As in Experiment 1, participants were not informed that some videos included gestures while others did not, allowing us to examine whether gestures naturally influenced their choices.

#### 3.1.4 Statistical analysis

We fitted a Bayesian multinomial logistic regression with a categorical family and logit link using the brms package (Bürkner, 2017, 2018, 2021) in R version 2026.01.0 (R Core Team, 2024). Model 2 was specify to predict the probabilities of selecting the preferred, possible, or impossible interpretation as a function of gesture (gesture-of-preferred, gesture-of-possible, non-gestured) and familiarity, with varying intercepts for participants and items and item-specific varying slopes for gesture:


$$\begin{array}{ccc} \text{choice} & \sim & \text{gesture}+\text{familiarity}+\left(1|\text{participant}\right)\\ +\left(1+\text{gesture}|\text{item}\right) \end{array}$$

Model 2a additionally examined whether the effect of gesture varied as a function of familiarity by including the gesture × familiarity interaction:


$$\begin{array}{ccc} \text{choice} & \sim & \text{gesture}*\text{familiarity}+\left(1|\text{participant}\right)\\ +\left(1+\text{gesture}|\text{item}\right) \end{array}$$

Models 2 and 2a used weakly informative priors, *N*(0, 1), for all regression coefficients. All models converged well ($\hat{R}=1.0$), and posterior predictive checks indicated that the models accurately captured the distribution of the observed data.

### 3.2 Results

This section first presents descriptive statistics for the forced-choice task, followed by the regression analysis examining whether gestures influenced metaphor comprehension. The probabilities of choosing each interpretation (preferred, possible, impossible) were modeled as a function of gesture (gesture-of-preferred, gesture-of-possible, non-gestured).

If gestural information can shift an individual's default interpretation, the probability of choosing the possible interpretation should be higher than that of choosing the preferred interpretation in the gesture-of-possible condition. Conversely, if gestures do not shift the default interpretation, the probability of choosing the possible interpretation should be similar to or lower than that of choosing the preferred interpretation in the gesture-of-possible condition. Moreover, a higher probability of selecting the possible interpretation in the gesture-of-possible condition compared to the gesture-of-preferred condition would indicate that gestures modulate the default interpretation without fully shifting it.

#### 3.2.1 Descriptive statistics

Table 4 presents the proportion of trials on which each interpretation (preferred, possible, impossible) was selected as a function of gesture condition (gesture-of-preferred, gesture-of-possible, non-gestured).

**Table 4:** Proportion of preferred, possible, and impossible interpretations as a function of gesture.

Gesture	Preferred	Possible	Impossible
Gesture-of-preferred	0.82	0.10	0.09
Gesture-of-possible	0.56	0.38	0.06
Non-gestured	0.73	0.14	0.13

#### 3.2.2 Regression model

**Figure 3:**
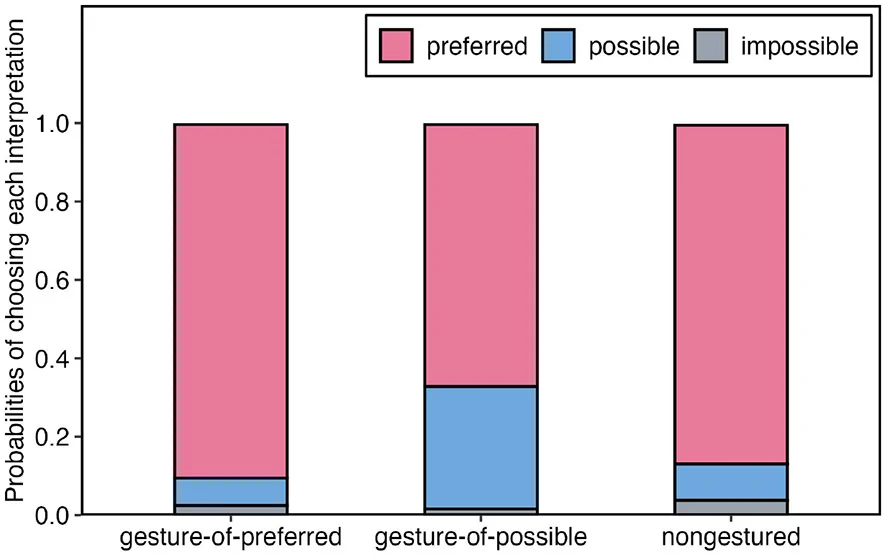
Predicted probabilities of choosing the preferred, possible, and impossible interpretations across levels of gesture from Model 2.

The regression coefficients from Model 2 are summarized in Table 5.^6^ Across all conditions, the preferred interpretation showed higher probabilities of being selected than both the possible and impossible interpretations (see Figure 3). However, the probability of selecting the possible interpretation increased in the gesture-of-possible condition compared to both the gesture-of-preferred condition ($\hat{\beta }=1.79$, 95% CrI[1.02, 2.52]) and the non-gestured condition ($\hat{\beta }=1.39$, 95% CrI[0.83, 1.99]). Moreover, the probability of selecting the preferred interpretation decreased from the gesture-of-preferred to the gesture-of-possible condition ($\hat{\beta }=-1.82$, 95% CrI[-2.54, -1.12]).

**Table 5:** Model 2 regression coefficients predicting the log-odds of choosing an interpretation by gesture.

Parameter	$\hat{\beta }$	95% CrI
Fixed effects (log-odds relative to preferred)		
*Possible vs. Preferred*		
Intercept	-2.67	[-4.44, -0.91]
Gesture (gesture-of-possible)	1.79	[1.01, 2.54]
Gesture (non-gestured)	0.31	[-0.40, 0.95]
Familiarity	0.03	[-0.44, 0.48]
*Impossible vs. Preferred*		
Intercept	-4.13	[-7.57, -0.75]
Gesture (gesture-of-possible)	-0.17	[-1.27, 0.77]
Gesture (non-gestured)	0.47	[-0.42, 1.17]
Familiarity	0.14	[-0.76, 1.00]
Random effects		
*Item*		
Possible intercept	1.33	[0.77, 2.12]
Possible gesture-of-possible slope	1.58	[0.96, 2.39]
Possible non-gestured slope	1.01	[0.29, 1.86]
Impossible intercept	2.41	[1.47, 3.83]
Impossible gesture-of-possible slope	1.20	[0.15, 2.55]
Impossible non-gestured slope	0.62	[0.03, 1.54]
*Participant*		
Possible intercept	0.50	[0.23, 0.76]
Impossible intercept	0.44	[0.04, 0.84]

Using the posterior predictive distribution obtained from Model 2, we estimated the probabilities of selecting each interpretation in the gesture-of-possible condition. The mean posterior probability for the preferred interpretation was 0.66, for the possible interpretation was 0.31, and for the impossible interpretation was 0.03, roughly corresponding to the colors pink, blue, and gray, respectively, in the middle bar in Figure 3. The posterior probability that the possible interpretation exceeded the preferred interpretation was 0.16, indicating that preferred remained the most likely interpretation in the gesture-of-possible condition.

#### 3.2.3 Follow-up analyses

Similar to Experiment 1, we also examined whether the effect of gesture on choice varied as a function of familiarity.

##### 3.2.3.1 Familiarity effects

Model 2a included familiarity as an interaction term rather than as a covariate, resulting in an interaction between gesture (gesture-of-preferred, gesture-of-possible, non-gesture) and familiarity (ranging from 1.7 to 5.9).

Similar to Experiment 1, the interactions were small, and the 95% credible intervals included zero (see Table 6), providing no clear evidence that the effect of gesture on interpretation choice varied reliably across levels of familiarity.

**Table 6:** Model 2a regression coefficients predicting the log-odds of choosing an interpretation by gesture and familiarity.

Parameter	$\hat{\beta }$	95% CrI
Fixed effects (log-odds relative to preferred)		
*Possible vs. Preferred*		
Intercept	-2.38	[-4.33, -0.32]
Gesture (gesture-of-possible)	1.20	[-0.38, 2.69]
Gesture (non-gestured)	0.16	[-1.19, 1.51]
Familiarity	-0.08	[-0.67, 0.45]
Gesture-of-possible × familiarity	0.21	[-0.25, 0.67]
Non-gestured × familiarity	0.06	[-0.33, 0.49]
*Impossible vs. Preferred*		
Intercept	-3.86	[-7.30, -0.32]
Gesture (gesture-of-possible)	-0.54	[-2.02, 1.02]
Gesture (non-gestured)	-0.02	[-1.29, 1.24]
Familiarity	0.03	[-0.93, 0.92]
Gesture-of-possible × familiarity	0.13	[-0.39, 0.58]
Non-gestured × familiarity	0.16	[-0.20, 0.50]
Random effects		
*Item*		
Possible intercept	1.40	[0.80, 2.24]
Possible gesture-of-possible slope	1.66	[1.01, 2.48]
Possible non-gestured slope	1.05	[0.21, 1.90]
Impossible intercept	2.46	[1.51, 3.92]
Impossible gesture-of-possible slope	1.25	[0.15, 2.80]
Impossible non-gestured slope	0.58	[0.03, 1.51]
*Participant*		
Possible intercept	0.50	[0.26, 0.76]
Impossible intercept	0.46	[0.05, 0.86]

### 3.3 Discussion

Experiment 2 examined whether participants integrate gestural information into their judgments when required to select a single interpretation. Choice was modeled as a function of whether the accompanying gesture matched the preferred interpretation, the possible interpretation, or whether no gesture was present. Across all gesture conditions, participants were more likely to select the default (preferred) interpretation, even when the gesture matched the possible interpretation. However, the likelihood of choosing the possible interpretation increased when the gesture matched the possible interpretation. These results are consistent with Experiment 1, showing that co-speech gestures influence metaphor comprehension, but listeners still favor default interpretations. When participants could evaluate both the preferred and possible interpretations, both received similar ratings in the gesture-of-possible condition. In contrast, when participants were forced to choose only one interpretation, they typically selected the default interpretation, even when the speaker gestured another plausible interpretation.

Experiments 1 and 2 examined how information conveyed through gestures influences the way metaphors are interpreted. However, it remains unclear whether this influence is bidirectional. Experiment 3 investigated whether, similarly to how gestures modulate metaphor comprehension, verbal content can also influence the way gestures are interpreted.

## 4 Experiment 3: gesture evaluation task

The goal of Experiment 3 was to investigate whether the interpretation of gestures is similarly influenced by speech cues. We examined whether the interpretation of the same gesture was modulated by the multimodal cues that were presented with it. To this end, the videos used in Experiments 1 and 2 were modified for presentation in three multimodal conditions. These conditions included no additional cues (muted audio, blurred lips), lip movements only (muted audio, visible lips), and full speech cues (audible audio, visible lips), with participants assigned to one condition only.

### 4.1 Method

#### 4.1.1 Participants

A total of 150 native English speakers (aged 21–70 years, M = 41.98, SD = 12.03; 84 females) were recruited via Prolific. Participants registered for one of three versions of the study, with 50 participants per group.^7^ The study lasted approximately 10 minutes, and participants received £2.50. All participants were born in and were currently residing in the United States.

#### 4.1.2 Materials

**Figure 4:**
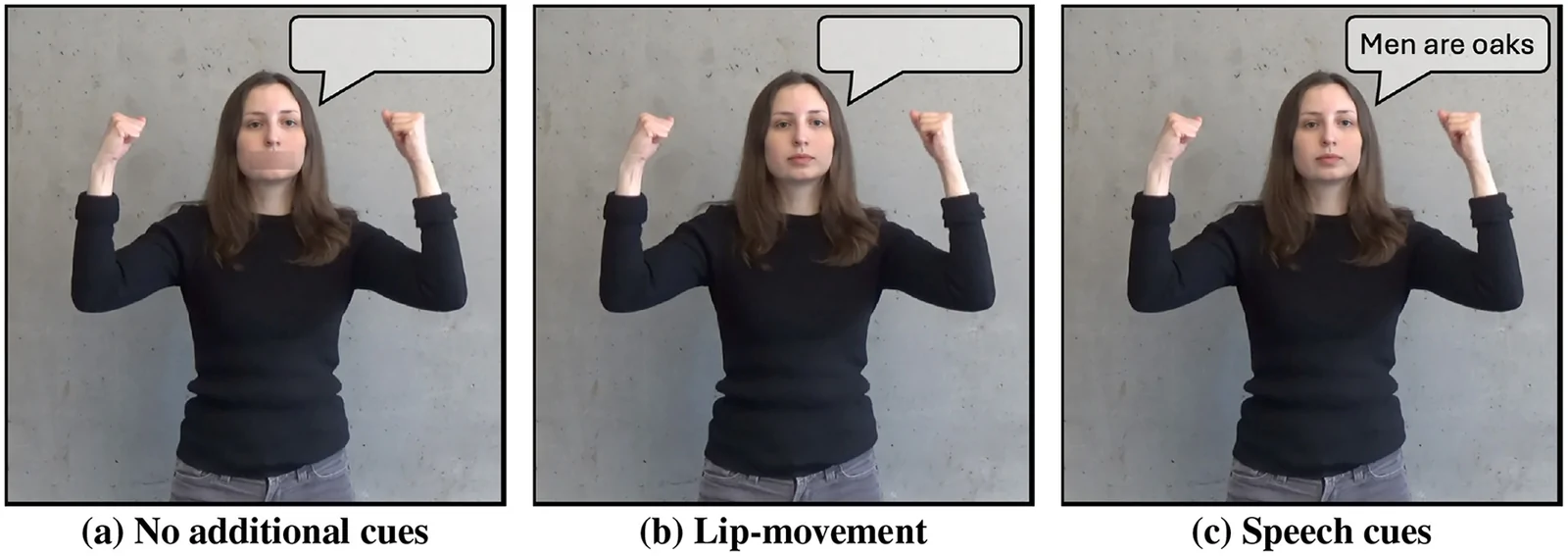
Video frames showing the same “robust” gesture across the three multimodal conditions. **(a)** No additional cues. **(b)** Lip-movement. **(c)** Speech cues.

We used the video clips from the gesture norming in Experiment 1 as a baseline and created two additional versions of each clip to examine the effects of multimodal cues. In the baseline condition with no additional cues, participants watched the clip with muted audio and blurred lips (Figure 4a). In the lip-movement condition, participants watched the clip with muted audio and visible lips (Figure 4b). In the speech condition, participants watched the clip with visible lips and audible speech (Figure 4c). Note that the two gestures are independent of each other, such that the “robust” gesture does not imply tall stature, and vice versa. Thus, a high rating for the interpretation *robust* after viewing the ‘robust' gesture does not imply a high rating for the interpretation *tall*.

The gestured interpretation (e.g., *robust*) corresponded directly to the gesture. The alternative interpretation (e.g., *tall*) was the other interpretation of the metaphor but was unrelated to the gesture. The unrelated interpretation (e.g., *hikers*) was not associated with either the gesture or the speech (see Supplementary Table 1).

#### 4.1.3 Procedure

The procedure was identical to that of the gesture norming study in Experiment 1. Participants watched each video and subsequently rated how well the gesture corresponded to each interpretation (gestured, alternative, unrelated) using a five-point meaningfulness scale. Participants completed 24 critical trials and four attention trials and had access to a SKIP button. The multimodal condition was implemented between groups, such that each participant viewed videos with either no additional cues, only lip movements, or speech cues. The interpretations were implemented within subjects, such that all participants rated all three interpretations.

#### 4.1.4 Statistical analysis

We fitted Bayesian ordinal regression models with a cumulative family and a logit link using the brms package (Bürkner, 2017, 2018, 2021) in R version 2026.01.0 (R Core Team, 2024). Model 3 was specified to predict changes in meaningfulness ratings as a function of multimodal cues (no additional cues, lip movement, speech cues) and interpretation (gestured, alternative, unrelated), with varying intercepts for participants and items:


$$\begin{array}{ccc} \text{rating} & \sim & \text{multimodal cues}*\text{interpretation}+\left(1|\text{participant}\right)\\ +\left(1|\text{item}\right) \end{array}$$

Model 3a examined whether the effect of multimodal cues varied as a function of preference, restricted to the subset of gestured interpretations:


$$\begin{array}{ccc} \text{rating} & \sim & \text{multimodal cues}*\text{preference}+\left(1|\text{participant}\right)\\ +\left(1|\text{item}\right) \end{array}$$

Model 3b examined whether the effect of multimodal cues varied as a function of relatedness, restricted to the subset of unrelated interpretations:


$$\begin{array}{ccc} \text{rating} & \sim & \text{multimodal cues}*\text{relatedness}+\left(1|\text{participant}\right)\\ +\left(1|\text{item}\right) \end{array}$$

Models 3, 3a, and 3b used weakly informative priors, *N*(0, 1), for all regression coefficients. All models converged well ($\hat{R}=1.0$), and posterior predictive checks indicated that the models accurately captured the distribution of the observed data.

### 4.2 Results

This section reports descriptive statistics for the gesture evaluation task, followed by the results of regression models examining the effects of multimodal cues (no additional cues, lip-movement, speech cues) and interpretation (gestured, alternative, and unrelated) on meaningfulness ratings.

We hypothesized that adding speech cues would increase the meaningfulness of the alternative interpretation, related to the metaphor in the speech but not to the gesture itself. Specifically, if participants see the “robust” gesture with no speech cues, we expect ratings for the gestured interpretation (e.g., *robust*) to be highest, and ratings for the alternative (e.g., *tall*) and unrelated (e.g., *hikers*) interpretations to be lowest. However, if participants see the “robust” gesture with speech cues (e.g., participants hear *Men are oaks*), we expect ratings for the alternative interpretation (e.g., *tall*) to increase, as men being tall is a plausible meaning of the metaphor.

We also expected that adding speech cues would slightly increase the meaningfulness of the gestured interpretation, since the content of the speech is supportive of the gesture, it may increase confidence. In contrast, we expected that ratings for the unrelated interpretation would not vary with the addition of speech cues, as they are unsupported by both the gesture and the speech. Moreover, the intermediate lip-movement condition was included to explore whether participants can extract speech content from visible lip movements in the absence of audible speech.

#### 4.2.1 Descriptive statistics

In the baseline condition, gestured interpretations received higher meaningfulness ratings (M = 3.87, SD = 1.34) than both alternative interpretations (M = 1.96, SD = 1.24) and unrelated interpretations (M = 1.65, SD = 1.07). These results indicate that the gestures were readily understandable even in the absence of additional multimodal cues.

In all attention trials (i.e., gestures of a heart, a thumb, the numbers three and four), the gestured interpretations received and the highest ratings (M = 4.94, SD = 0.40) compared with the alternative (M = 1.07, SD = 0.39) and unrelated interpretations (M = 1.04, SD = 0.31). Therefore, no participants were excluded based on these attention checks, as all rated the gestured interpretations at *M*≥4.00 and the alternative or unrelated interpretations at *M* ≤ 2.25. The SKIP button was used only eight times (0.02% of trials) in the entire study.

#### 4.2.2 Regression model

**Figure 5:**
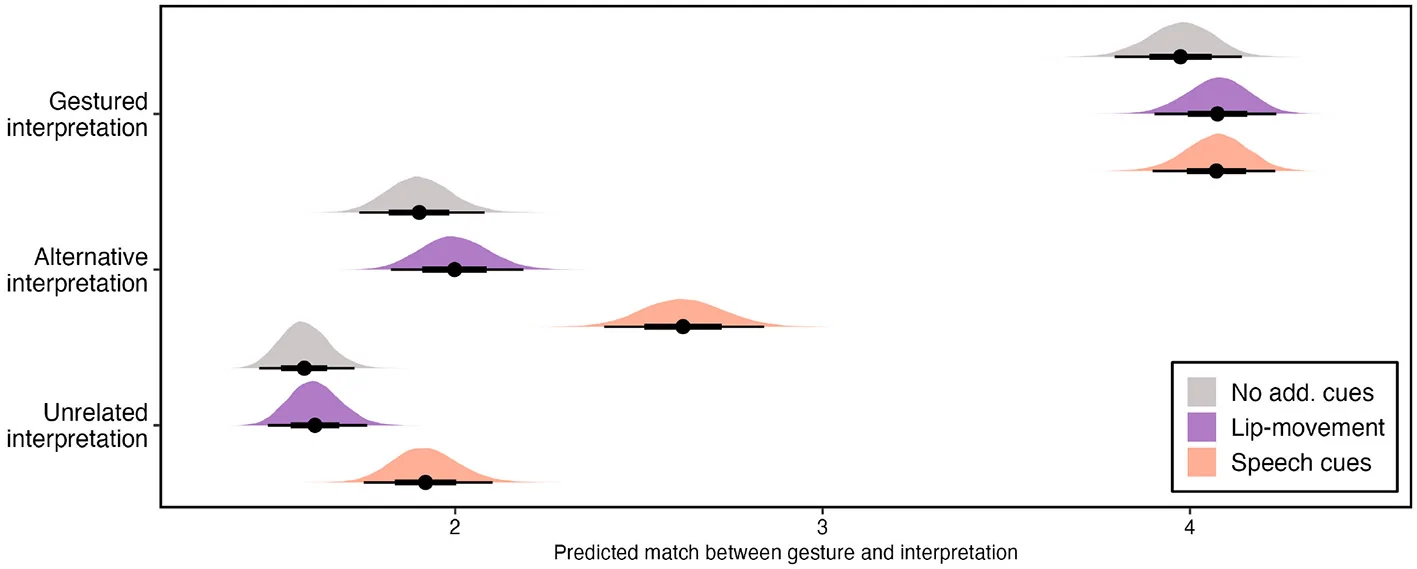
Meaningfulness ratings for the gestured interpretation, alternative interpretation of the metaphor, and unrelated interpretation across multimodal conditions.

The regression coefficients from Model 3 are summarized in Table 7. We observed a main effect of interpretation, with gestured interpretations receiving overall higher meaningfulness ratings than both alternative and unrelated interpretations (see Figure 5). We also observed an interaction between multimodal cues and interpretation, whereby the alternative interpretation was more likely to receive higher ratings when presented with speech cues than when presented with no additional cues (β = 0.84, 95% CrI[0.62, 1.06]) or with only lip movements (β = 0.85, 95% CrI[0.65, 1.06]), while the gestured interpretation received similar ratings across all multimodal conditions (see Figure 5).

**Table 7:** Model 3 regression coefficients predicting the log-odds of higher meaningfulness ratings by multimodal cues and interpretation.

Parameter	$\hat{\beta }$	95% CrI
Fixed effects		
Alternative interpretation	−2.89	[−3.06, −2.73]
Unrelated interpretation	−3.49	[−3.66, −3.32]
Lip-movement	0.19	[−0.13, 0.50]
Speech cues	0.20	[−0.12, 0.51]
Alternative × Lip-movement	−0.04	[−0.26, 0.17]
Unrelated × Lip-movement	−0.14	[−0.37, 0.09]
Alternative × Speech	0.84	[0.62, 1.06]
Unrelated × Speech	0.41	[0.19, 0.64]
Random effects		
*Item intercept*	0.45	[0.33, 0.61]
*Participant intercept*	0.71	[0.62, 0.80]

Interestingly, the unrelated interpretation was also more likely to receive higher ratings when presented with speech cues than when presented with no additional cues (β = 0.41, 95% CrI[0.19, 0.64]) or with only lip movements (β = 0.47, 95% CrI[0.24, 0.69]), though to a lesser extent compared to the alternative interpretation.

Lastly, for none of the interpretations did the lip-movement condition differ from the condition with no additional cues (see Figure 5).

#### 4.2.3 Follow-up analyses

The following analyses examined whether the absence of the effect of speech cues for gestured interpretations reflected properties of the metaphors themselves, and whether the unexpected effect of speech cues for unrelated interpretations was driven by the semantic relatedness between those interpretations and individual words in the speech.

##### 4.2.3.1 Gestured interpretations

To investigate why ratings for the gestured interpretation did not differ across multimodal conditions, we examined whether these ratings depended on how metaphors were interpreted. Since each metaphor had two plausible interpretations, one preferred and one less preferred but still possible, ratings could vary depending on whether the gesture matched the preferred or possible one. To assess this, we examined whether the effect of multimodal cues depended on preference.

**Figure 6:**
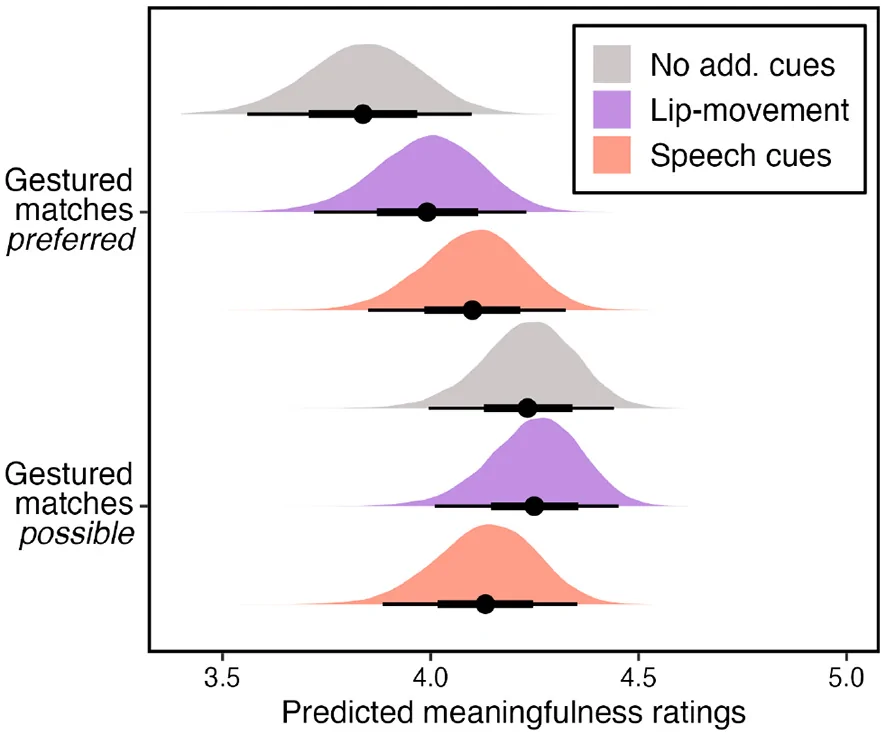
Meaningfulness ratings for gestured interpretations when they matched the preferred versus possible interpretations of the metaphor across multimodal conditions.

Model 3a used the same model specification as Model 3 to predict changes in meaningfulness ratings for the gestured interpretations as a function of preference, multimodal cues, and their interaction. We observed an interaction between preference and multimodal cues. When the gestured interpretation matched the preferred interpretation of the metaphor, it was more likely to receive higher ratings when paired with speech cues compared to no additional cues (β = 0.45, 95% CrI[0.06, 0.84]). In contrast, when the gestured interpretation matched the possible interpretation of the metaphor, ratings did not differ between speech cues and no additional cues (β = −0.18, 95% CrI[−0.58, 0.22]). Thus, the effect of speech cues was observed for the gestured interpretation only when it matched the preferred interpretation of the metaphor, but not when it matched the possible one. In Figure 6, this difference is visible by comparing speech (orange) and no additional cues (gray) in each sublevel of the gestured interpretation.

##### 4.2.3.2 Unrelated interpretations

To investigate why ratings for the unrelated interpretation increased in the speech condition, even though these interpretations were not associated with the speech, we examined whether the ratings depended on the semantic relationship between the unrelated interpretation and the individual words in the utterance. We computed cosine similarities between Word2Vec word embeddings^8^ for the unrelated interpretation (e.g., *hikers*) and both the topic (e.g., *men*) and vehicle (e.g., *oaks*) of each metaphor, separately. Cosine similarity measures the semantic distance between word embeddings, with higher values indicating greater semantic relatedness. Items for which the unrelated interpretation had a similarity greater than 0.25 with either the topic or vehicle were coded as *literally related*, and all others as *truly unrelated*. This procedure yielded 12 literally related interpretations (e.g., *sticky* relative to *glue*) and 12 truly unrelated interpretations (e.g., *square* relative to *cotton*).

**Figure 7:**
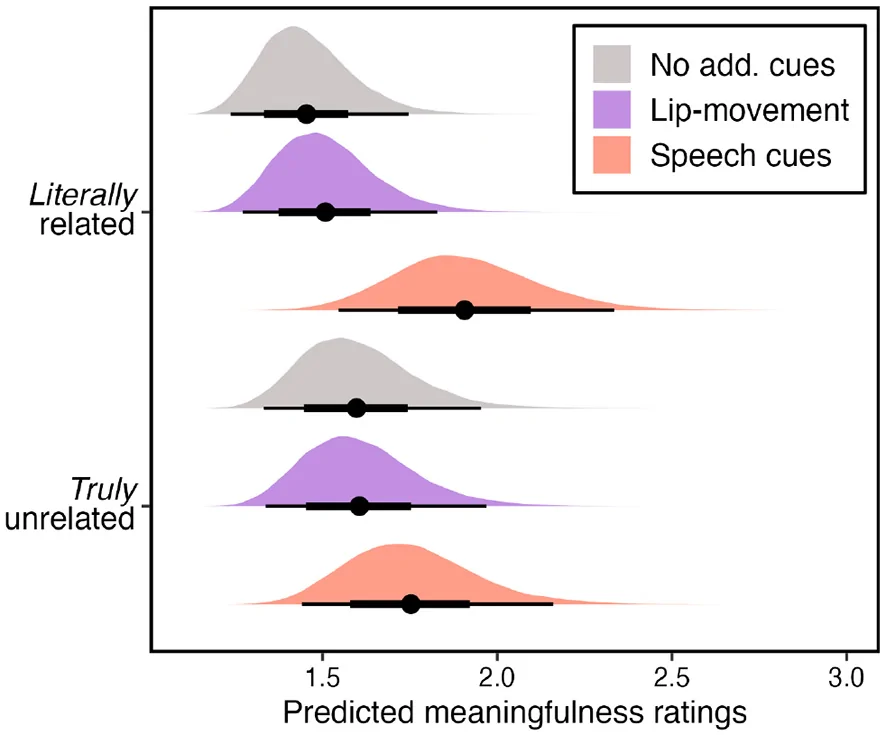
Meaningfulness ratings for unrelated interpretations when they were literally related versus truly unrelated to the metaphor across multimodal conditions.

Model 3b used the same model specification as Model 3 to predict changes in meaningfulness ratings for the unrelated interpretations as a function of relatedness, multimodal cues, and their interaction. We observed an interaction between relatedness and multimodal cues. When the unrelated interpretation was literally related to the individual words in the speech, it was more likely to receive higher ratings when presented with speech cues compared to no additional cues (β = 0.94, 95% CrI[0.49, 1.40]). In contrast, when the unrelated interpretation was truly unrelated to the individual words in the speech, ratings did not differ between speech cues and no additional cues (β = 0.34, 95% CrI[−0.11, 0.79]). Thus, the effect of speech cues was only present for the literally related subset, but not for the truly unrelated subset. In Figure 7, this difference is also visible by comparing speech (orange) and no additional cues (gray) in each sublevel of the unrelated interpretation.

### 4.3 Discussion

Experiment 3 examined how gesture comprehension is integrated with multimodal cues by presenting videos of the same gesture with no additional cues, with only lip movements, and with audible speech. We specifically assessed whether meaningfulness ratings for gestured, alternative, and unrelated interpretations varied with the addition of multimodal cues. The gestured interpretation reflected the meaning conveyed by the gesture, the alternative interpretation reflected a plausible meaning of the metaphor in the speech but was unrelated to the gesture, and the unrelated interpretation was independent of both speech and gesture.

As predicted, the perceived meaningfulness of the alternative interpretation increased when the gesture was paired with speech cues. This pattern is particularly interesting because it shows that participants incorporated the alternative figurative sense of the metaphor even when it was unnecessary for assessing the gesture. When the actor performed the ‘robust' gesture while saying *Men are oaks*, ratings for the gestured interpretation (*robust*) remained high and stable across conditions, whereas ratings for the alternative interpretation (*tall*) increased, despite *tall* being unrelated to the observed gesture.

These findings indicate that participants not only attended to the gesture, but also integrated it with both figurative senses of the utterance. In contrast, the perceived meaningfulness of the gestured interpretation did not change when speech cues were added, initially suggesting that when a gesture is clear, supportive verbal content does not further enhance comprehension. However, when the preferred and possible interpretations of the metaphor were differentiated, an effect of speech cues emerged for gestured interpretations that matched the preferred interpretation. These results suggest that participants were sensitive to the dominant figurative sense of the metaphor in the speech, which, when aligned with the gesture, contributed to the observed effect.

Surprisingly, the perceived meaningfulness of the unrelated interpretation also increased when presented with speech cues, although to a lesser extent than the alternative interpretation. This may reflect participants' sensitivity to aspects of the speech, including both figurative and literal content. Since these materials were designed to study metaphor comprehension, some unrelated interpretations could be literally associated with the metaphor. For example, as shown in Supplementary Table 1, the unrelated interpretations *poisonous* and *sticky* could apply literally to a snake and glue, respectively, but not to the figurative senses of *Roads are snakes* or *Trust is glue*. Similarly, *informative* and *hurtful* could describe schools and people, respectively, but are not derived from the figurative senses of *Schools are zoos* or *People are arrows*. Indeed, the effect of speech cues, previously observed for alternative interpretations, was present for literally related interpretations but absent for truly unrelated ones. This suggests that gesture comprehension was influenced not only by the figurative content of the metaphor but also by the literal meaning of the metaphor in the speech.

In sum, the effect of speech cues was present for *all* interpretations in specific situations: (1) for gestured interpretations, when they matched the dominant figurative sense of the spoken metaphor; (2) for alternative interpretations overall; and (3) for unrelated interpretations, when they were literally related to the spoken metaphor. We did not find an effect of lip-movement information for any interpretation, suggesting that individuals are not able to reconstruct verbal content from lip movements alone, at least this seems to be the case when the video frame includes the face and torso rather than just a close-up of the face and when no prior context is available to predict subsequent words.

## 5 General discussion

We investigated how speech and gesture combine during comprehension to derive meaning. Experiments 1 and 2 examined how the presence of gestures influenced metaphor comprehension, depending on whether they matched the preferred or a less preferred but still possible interpretation of the same expression. Across all gesture conditions, participants rated the preferred interpretation as most meaningful (Experiment 1) and selected it most often (Experiment 2). However, when gestures depicted the possible interpretation, meaningfulness ratings (Experiment 1) and probabilities of being selected (Experiment 2) increased significantly for the possible interpretation. Importantly, gestures modulated participants' responses even when gestures were not explicitly mentioned in the instructions, were not necessary to complete the task, and appeared in only half of the stimuli due to the inclusion of nongestured fillers. However, it remains possible that some participants became aware of the gesture manipulation during the experiment, which may have influenced their responses. Future studies could include awareness questions at the end of the experiment to evaluate this possibility.

These findings indicate that co-speech gestures modulated metaphor comprehension; however, gestures alone are insufficient to override default interpretations. One possible explanation is that, because the stimuli were brief and presented in a minimalist discourse context (i.e., without prior context), participants relied more strongly on the default interpretation of the utterance. In this case, speech-gesture integration is constrained by context. When contextual support is limited, the verbal modality is weighted more heavily due to its established preferred interpretations, thereby limiting the extent to which gestures, which do not have equally established preferred interpretations, can influence overall comprehension.

Another possibility is that, since we instructed the actor to perform the gestures in synchrony with the speech, the preparation phase generally coincided with the beginning of the utterance (e.g., *Men are*), the stroke of the gestures coincided with or occurred slightly after the onset of the vehicle (e.g., *oaks*), and the retraction of the hands extended slightly beyond the end of the utterance. But perhaps gestures had a greater impact if they had preceded rather than co-occurred with speech. As observed in imaging studies (Kelly et al., 2004), “gestures may create a visuospatial context that subsequently influences the sensory processing of the linguistic information that follows” (see also Habets et al., 2011). We plan to explore this next by manipulating the temporal relationship between speech and gesture using the same materials.

A third possibility is that, since we could not control whether all participants attended to the gestures—since explicitly directing attention might produce artificial responses and our goal was to examine whether gestures *spontaneously* influence comprehension—only a subset of participants may have actually viewed them, while others relied solely on the verbal modality. If so, future work could benefit from including individual difference measures or tracking eye movements to determine which participants tend to rely more on visual information and under what circumstances.

Experiment 3 examined how the presence of multimodal cues influenced gesture comprehension, depending on whether gestures were presented with no additional cues, only lip movements, or speech cues. We observed that when gestures were paired with speech cues, meaningfulness ratings for the alternative interpretation, related to the speech but not to the gesture, significantly increased compared to conditions with no speech cues. These results are consistent with findings reported by Kelly et al. (1999), in which the interpretation of deictic gestures was modulated by the accompanying speech. As noted earlier, since speech slightly preceded the stroke of the gesture, it appears that speech functioned as context for the gesture and thus influenced its interpretation.

Nevertheless, we cannot determine whether the integration of speech and gesture occurs automatically, as reported in some studies (e.g., Kelly et al., 2010a), under voluntary control, as reported in others (e.g., Holle and Gunter, 2007), or both under different circumstances. For instance, Kelly et al. (2004) observed that iconic gestures complementary to speech (e.g., when the target object was a glass, participants heard *tall* and saw a “thin” gesture) were integrated at early stages of comprehension, whereas gestures incongruent with speech (e.g., when the target object was a dish, participants heard *tall* and saw a ‘short' gesture) were integrated at both early and late stages. However, the metaphoric gestures used in the present study are neither complementary nor incongruent, as they align with an alternative viable interpretation of the utterance (henceforth, alternative gestures). Since our study measured offline responses, we did not evaluate early stages of processing, only post-semantic stages, in which information from alternative gestures modulated metaphor interpretation. In this sense, alternative gestures resemble incongruent gestures more than complementary gestures.

Moreover, given that in Experiment 3 both the literal meaning and figurative content of the metaphor influenced gesture interpretation, we consider another possibility. The literal meaning of metaphors is accessed before the figurative content and may linger further downstream (Pissani and de Almeida, 2022, 2023; Pissani et al., 2026; Weiland et al., 2014). Specifically, Pissani and de Almeida (2023) observed that maze choices were influenced by the literal meaning of preceding metaphors up to 13 words (roughly 3,250 ms) before the decision point. In the present study, concepts associated with the literal meaning of the metaphors could have influenced gesture interpretation at early stages, consistent with a more automatic process. However, given that our videos averaged approximately five seconds in duration (M = 5.23, SD = 0.57), it seems less likely that literal concepts would persist long enough to influence participants' responses.

Altogether, these findings support the integrated-systems hypothesis, which posits that speech and gesture mutually and obligatorily influence one another. In our study, alternative gestures modulated the interpretation of metaphors compared to conditions without gestures, and conversely, verbal content influenced the interpretation of gestures compared to conditions without additional multimodal cues. These bidirectional effects show that, even when gestures are not necessary to complete a task, they interact with speech to support comprehension.

## Author's note

Experiment 1 was preregistered. Experiments 1 and 2 were presented at the 39th Annual Conference on Human Sentence Processing. Experiment 3 is also reported in the proceedings of the 48th Annual Meeting of the Cognitive Science Society (Pissani and Demberg, 2026) and was presented at both the 48th Annual Meeting of the Cognitive Science Society and the 32nd Architectures and Mechanisms for Language Processing Conference.

## Data Availability

The datasets presented in this study can be found in online repositories. The names of the repository/repositories and accession number(s) can be found in the article/Supplementary material. The online repository can be accessed here: https://osf.io/cfk7j.

## Footnotes

1 Sample sizes were determined based on prior studies using similar designs (e.g., Kelly et al., 2010b) and practical constraints on data collection.

2 No formal screening for neurological, psychiatric, or sensory conditions was conducted. Eligibility was based on standard Prolific prescreening criteria.

3 See (Pissani 2026) for the video stimuli repository.

4 Three impossible interpretations (*comical, addictive*, and *deep*) were rated higher than their corresponding possible interpretations (see Supplementary Table 3). These items were not originally intended for inclusion; however, because the overall pattern of ratings remained unchanged, all items were retained in the analyses.

5 Model 1 is an ordinal regression in which the dependent variable, ratings, is treated as an ordered categorical variable. Coefficients estimate the probabilities of moving from one rating category to the next. In Table 2, the preferred interpretation and gesture-of-preferred condition serve as reference levels. For example, the slope for the possible interpretation reflects the log-odds of it receiving a higher rating compared to the preferred interpretation in the gesture-of-preferred condition. Other contrasts (e.g., gesture-of-possible vs. non-gestured) were obtained by refitting the model with different reference levels.

6 Model 2 is a multinomial regression with a three-level dependent variable (preferred, possible, impossible). Coefficients estimate the probabilities of choosing an interpretation relative to a reference category. In Table 5, the preferred interpretation and the gesture-of-preferred condition serve as reference levels. For example, in the possible vs. preferred contrast, the intercept reflects the log-odds of selecting the possible interpretation (relative to the preferred one) in the gesture-of-preferred condition. Other contrasts were obtained by refitting the model with different reference levels.

7 The group that watched the version of the videos without additional cues (*N* = 50) was the same as the group of participants in the gesture norming study. Thus, for Experiment 3, we recruited 100 additional participants.

8 Word2Vec embeddings (Mikolov et al., 2013a,b) are numerical vector representations of word types learned from a window of surrounding context words, such that words with similar meanings or grammatical roles have similar vectors (Meghdadi et al., 2025).
