# Supplementary material for: Bidirectional effects of speech and gesture: evidence from a novel levels-of-congruence paradigm
Source: Front Psychol. 2026 Aug 19;17:1851588. doi: 10.3389/fpsyg.2026.1851588 (PMC13533646; doi:10.3389/fpsyg.2026.1851588)
Supplement: Supplementary file 1 [file Supplementary_File_1.pdf]

SUPPLEMENTARY MATERIALS

Pilot study

In this task, you will be presented with 28 expressions and six questions about each of them. Please, read the instructions and the example below carefully:

Example: Insults are daggers

1. How familiar is this expression to you?
You should report the extent to which you have heard or read that expression in the past by clicking on a number between 1 (not familiar at all) and 5 (very familiar).

2. What does "daggers" mean in this context?
You should write an adjective that best represents the meaning of the word in quotations in the expression above. For instance, an adjective that can capture the meaning of "daggers" in the expression above is "hurtful". If you have never heard the expression before, make your best guess.

3. Could "daggers" also mean "agonizing" in this context?
You should report how well you think "agonizing" conveys the intended meaning of "daggers" in the sentence above by clicking on a number between 1 (not at all) and 5 (very much).

4. Could "daggers" also mean "stinging" in this context?
You should report how well you think "stinging" conveys the intended meaning of "daggers" in the sentence above by clicking on a number between 1 (not at all) and 5 (very much).

5. What is a feature you associate with "insults"?
You should write an adjective that you associate with the word in quotations. For instance, an adjective that can be associated with "insults" is "mean".

6. What is a feature you associate with "daggers"?
You should write an adjective that you associate with the word in quotations. For instance, an adjective that can be associated with "daggers" is "sharp".

Gesture norms

Table S1. Example of materials used in the gesture norming study and in the gesture evaluation task, grouped by preference.

Gesture description	Gestured	Alternative	Unrelated	Metaphor
Gesture for the preferred interpretation of the metaphor				
Hand in a curvy motion ahead	WINDING	LONG	POISONOUS	*Roads are snakes*
One hand up, other strikes it	DRIVEN	FAST	HURTFUL	*People are arrows*
Fist hitting palm	DESTRUCTIVE	NOISY	HEAVY	*Salesmen are bulldozers*
Fingers circling temples	CRAZY	SMELLY	INFORMATIVE	*Schools are zoos*
Hands interlocking fingers	BONDING	STRONG	STICKY	*Trust is glue*
Gesture for the possible interpretation of the metaphor				
Hand signaling ahead	LONG	WINDING	POISONOUS	*Roads are snakes*
Hands sliding past each other	FAST	DRIVEN	FAST	*People are arrows*
Hands cupped around mouth	NOISY	DESTRUCTIVE	HEAVY	*Salesmen are bulldozers*
Hands fanning nose	SMELLY	CRAZY	INFORMATIVE	*Schools are zoos*
Hands clasped tightly	STRONG	BONDING	STICKY	*Trust is glue*

Table S2. Mean ratings (SD) of how well each gesture matched the gestured interpretation of a metaphor, compared to an alternative interpretation (associated with the metaphor but not gestured) and an unrelated interpretation (not associated with the gesture or the metaphor).

Metaphor	Interpretation	Gestured	Alternative	Unrelated
Anger is fire	destructive	2.1 (1.3)	1.3 (0.7)	1.3 (0.7)
	heat	3.4 (1.3)	1.4 (0.6)	2.1 (1.1)
Babies are roosters	noisy	4.9 (0.3)	1.1 (0.3)	1.1 (0.3)
	early risers	3.9 (1.4)	1.2 (0.4)	1.7 (1.0)
Christ is a door	open	4.5 (0.9)	2.0 (1.0)	1.0 (0.2)
	pathway	4.2 (1.1)	3.1 (1.3)	1.3 (0.7)
Cigarettes are time bombs	inevitable	3.1 (1.3)	1.2 (0.7)	1.3 (0.8)
	deadly	3.9 (1.3)	1.8 (1.2)	1.3 (0.5)
Clouds are cotton	fluffy	3.3 (1.4)	3.1 (1.2)	1.1 (0.3)
	soft	4.1 (1.1)	2.2 (1.4)	1.1 (0.3)
Deserts are ovens	hot	4.8 (0.8)	1.7 (0.9)	1.2 (0.6)
	dry	3.8 (1.5)	2.3 (1.3)	1.4 (0.7)
Doctors are gods	good	3.1 (1.2)	1.2 (0.5)	4.3 (0.9)
	powerful	3.4 (1.3)	2.1 (1.3)	2.0 (1.2)
Education is a journey	process	4.2 (1.0)	1.4 (0.9)	1.7 (1.1)
	hard	3.7 (1.4)	1.6 (0.9)	1.6 (0.9)
Engineers are squares	rigid	3.2 (1.7)	1.4 (0.8)	3.1 (1.3)
	smart	4.6 (0.9)	1.4 (0.8)	1.8 (1.1)
Family is a fortress	supportive	3.1 (1.5)	1.3 (0.7)	1.4 (0.8)
	large	4.7 (0.9)	1.8 (1.2)	1.4 (0.6)
Knowledge is a river	flowing	4.4 (1.0)	2.1 (1.1)	1.6 (0.9)
	long	3.5 (1.2)	3.3 (1.4)	1.2 (0.6)
Lawyers are sharks	strategic	4.2 (1.1)	1.8 (1.2)	1.5 (1.0)
	dangerous	1.8 (0.9)	2.3 (1.5)	2.1 (1.5)
Life is a joke	brief	4.0 (1.1)	1.5 (0.8)	1.4 (0.7)
	unserious	3.8 (1.1)	2.1 (1.4)	2.1 (1.2)
Love is a drug	powerful	4.4 (1.2)	1.3 (0.7)	1.2 (0.6)
	intoxicating	4.0 (0.9)	1.9 (0.9)	2.1 (1.1)
Memory is a sponge	absorbent	3.1 (1.5)	2.0 (1.3)	1.6 (0.9)
	expandable	4.8 (0.5)	1.8 (1.1)	1.6 (1.0)
Men are oaks	tall	5.0 (0.0)	1.6 (1.0)	1.4 (1.1)
	robust	4.4 (1.0)	1.8 (1.1)	1.4 (0.9)
People are arrows	driven	2.5 (1.2)	2.2 (1.2)	2.6 (1.4)
	fast	4.4 (0.7)	2.8 (1.4)	1.7 (0.9)
Pets are kids	loving	4.4 (1.0)	2.5 (1.2)	1.4 (0.7)
	innocent	3.2 (1.4)	3.4 (1.3)	1.5 (0.8)
Roads are snakes	long	3.9 (1.3)	1.3 (0.6)	1.0 (0.0)
	winding	5.0 (0.2)	2.6 (1.2)	1.5 (0.9)
Salesmen are bulldozers	destructive	4.4 (0.8)	1.5 (0.8)	2.2 (1.1)
	noisy	3.8 (1.1)	1.7 (1.0)	1.2 (0.4)
Schools are zoos	crazy	4.5 (1.1)	1.1 (0.4)	1.7 (1.2)
	smelly	4.9 (0.4)	1.4 (0.7)	1.2 (0.6)
Trust is glue	strong	3.1 (1.7)	4.3 (0.7)	2.2 (1.3)
	bonding	4.7 (0.6)	2.9 (1.3)	2.2 (1.2)
Wisdom is an ocean	powerful	2.4 (1.3)	2.6 (1.4)	2.6 (1.4)
	vast	4.6 (0.6)	1.9 (0.9)	1.6 (0.9)
Women are roses	fragrant	3.9 (1.0)	1.6 (0.9)	1.1 (0.3)
	beautiful	2.8 (1.5)	2.4 (1.4)	1.3 (0.5)

Metaphor norms

Table S3. Familiarity ratings using non-gestured videos, along with meaningfulness ratings for the preferred, possible, and impossible interpretations using non-gestured videos from Experiment 1.

Metaphor	Familiarity	Preferred	M (SD)	Possible	M (SD)	Impossible	M (SD)
Anger is fire	4.0 (2.2)	destructive	4.2 (1.1)	heat	3.5 (1.1)	colorful	1.4 (0.7)
Babies are roosters	1.6 (1.1)	early risers	4.1 (0.9)	noisy	3.8 (1.2)	animalistic	2.1 (1.4)
Christ is a door	2.5 (1.9)	pathway	4.1 (1.3)	open	3.6 (1.3)	locked	1.7 (1.2)
Cigarettes are time bombs	3.3 (2.1)	deadly	4.8 (0.5)	inevitable	3.5 (1.5)	cool	1.0 (0.0)
Clouds are cotton	3.8 (2.1)	fluffy	4.3 (0.9)	soft	4.0 (1.3)	square	1.3 (0.8)
Deserts are ovens	3.7 (1.9)	hot	4.8 (0.7)	dry	4.0 (1.3)	empty	1.9 (1.1)
Doctors are gods	2.6 (1.8)	powerful	4.4 (0.8)	good	2.6 (1.2)	generous	2.4 (1.4)
Education is a journey	5.2 (1.8)	process	4.3 (0.8)	hard	2.7 (1.1)	rudimentary	2.0 (1.2)
Engineers are squares	2.9 (2.0)	rigid	3.5 (1.3)	smart	3.2 (1.2)	structured	3.5 (1.1)
Family is a fortress	3.1 (1.9)	supportive	4.0 (1.2)	large	2.6 (1.3)	weird	1.4 (0.9)
Knowledge is a river	3.2 (2.1)	flowing	4.2 (0.9)	long	3.0 (1.4)	wet	1.6 (1.3)
Lawyers are sharks	5.4 (1.9)	dangerous	3.5 (1.3)	strategic	3.4 (1.2)	hungry	2.1 (1.2)
Life is a joke	4.8 (2.0)	unserious	4.0 (0.8)	brief	2.0 (1.3)	comical	4.0 (0.9)
Love is a drug	5.9 (1.6)	intoxicating	4.2 (1.3)	powerful	3.6 (1.4)	addictive	4.4 (0.9)
Memory is a sponge	4.6 (2.1)	absorbent	4.4 (0.9)	expandable	3.6 (0.9)	smooth	1.5 (0.9)
Men are oaks	2.4 (1.6)	robust	3.6 (1.3)	tall	3.4 (1.3)	hikers	1.3 (0.9)
People are arrows	1.7 (1.3)	driven	3.4 (1.4)	fast	2.7 (1.4)	hurtful	2.5 (1.6)
Pets are kids	4.8 (1.8)	innocent	3.9 (1.2)	loving	3.6 (1.2)	small	3.1 (1.1)
Roads are snakes	2.6 (1.9)	winding	4.3 (1.1)	long	3.6 (1.4)	poisonous	1.4 (0.6)
Salesmen are bulldozers	2.2 (1.6)	destructive	3.3 (1.3)	noisy	2.2 (1.4)	heavy	2.2 (1.5)
Schools are zoos	3.8 (2.1)	crazy	3.6 (1.5)	smelly	2.5 (1.1)	informative	1.8 (0.8)
Trust is glue	3.0 (2.1)	bonding	4.4 (0.8)	strong	4.0 (1.2)	sticky	2.5 (1.6)
Wisdom is an ocean	3.0 (1.9)	vast	4.6 (0.8)	powerful	3.2 (1.6)	deep	4.4 (0.6)
Women are roses	3.3 (2.0)	beautiful	4.6 (0.8)	fragrant	3.0 (0.9)	red	1.6 (1.0)
